# Pre-Matching Circuit for High-Frequency Ultrasound Transducers

**DOI:** 10.3390/s22228861

**Published:** 2022-11-16

**Authors:** Hojong Choi

**Affiliations:** Department of Electronic Engineering, Gachon University, Seongnam 13120, Republic of Korea; hojongch@gachon.ac.kr

**Keywords:** high-frequency ultrasound transducer, pre-matching circuit, ultrasound instrument

## Abstract

High-frequency ultrasound transducers offer higher spatial resolution than low-frequency ultrasound transducers; however, their maximum sensitivity are lower. Matching circuits are commonly utilized to increase the amplitude of high-frequency ultrasound transducers because the size of the piezoelectric material decreases as the operating frequency of the transducer increases. Thus, it lowers the limit of the applied voltage to the piezoelectric materials. Additionally, the electrical impedances of ultrasound transducers generally differ at the resonant-, center-, and anti-resonant-frequencies. The currently developed most-matching circuits provide electrical matching at the center frequency ranges for ultrasound transmitters and transducers. In addition, matching circuits with transmitters are more difficult to use to control the echo signal quality of the transducers because it is harder to control the bandwidth and gain of an ultrasound transmitter working in high-voltage operation. Therefore, we provide a novel pre-matching circuit method to improve the amplitude and bandwidth of high-frequency ultrasound transducers at the resonant-, center-, and anti-resonant-frequency ranges, with an ultrasound receiver and transducer. To verify the pre-matching circuit, pulse-echo response tests were conducted on the ultrasound transducers. The results show that the designed pre-matching circuits provide higher amplitude (5.63- and 2.02-times) and wider bandwidth (175.55% and 62.01%) for the high-frequency ultrasound transducer compared to the original circuit without a pre-matching circuit, and the parallel capacitor with a series-inductor circuit, respectively; therefore, the proposed pre-matching circuit is an appropriate solution for improving the amplitudes and bandwidths of high-frequency ultrasound transducers over wide frequency ranges.

## 1. Introduction

Ultrasound has been utilized for non-destructive testing, imaging, acoustic trapping, and therapeutic instruments [1,2,3,4]. Compared to low-frequency (<15 MHz) ultrasound, high-frequency (≥15 MHz) ultrasound offers higher spatial resolutions while scarifying the target sensitivity for the same triggering power [5,6]. Owing to these advantages, high-frequency ultrasound has been recently highlighted for applications in dermatology, ophthalmology, small animal imaging, intravascular ultrasound, acoustic tweezers, and acoustic cell sorters [7,8,9,10].

Ultrasound instruments are composed of ultrasound transmitters, receivers, and transducers [11]. The last and first stages of the front-end circuits of ultrasound transmitters and receivers are power amplifiers, preamplifiers, and limiters [12]; the power amplifiers trigger the ultrasound transducers, the preamplifiers amplify the echo signals received from the ultrasound transducers, and the limiters block unwanted high-voltage signals from the power amplifiers [1]. These components are highly related to the performance of ultrasound instruments.

Unfortunately, the performance of high-frequency ultrasound transducers, which are some of the most important components in ultrasound instruments, is typically lower than that of low-frequency ultrasound transducers [13]; therefore, the sensitivity of high-frequency ultrasound transducers is limited because the size of the piezoelectric material is inversely proportional to the frequency of the ultrasound transducer [1,5]. As the frequency of the ultrasound transducers increases, the maximum applied voltages generated by the ultrasound transmitters that trigger the ultrasound transducers should decrease because the maximum tolerable voltage is proportional to the size of the piezoelectric material [13,14]. Therefore, matching circuits must be utilized to improve the performance of high-frequency ultrasound transducers.

Most matching circuits used in ultrasound instruments are integrated with ultrasound transmitters, which limit the components’ maximum voltage and possibly the transmitters’ performance [15,16,17]. The matching circuits typically use a series capacitor with a parallel inductor, a series inductor with a parallel capacitor, or a Pi- or T-network that are composed of the capacitors and inductors [18,19,20]. Currently developed matching circuits utilize a matching function with the ultrasound transmitter as it is more difficult to control the amplitude and bandwidths of the echo signals generated by the ultrasound transducers. However, an ultrasound receiver working in a low-voltage operation, e.g., bandwidth and gain, is easier to control externally than an ultrasound transmitter working in a high-voltage operation [15,21,22]; therefore, we designed a matching circuit for an ultrasound receiver. It offers the freedom of component selection for the maximum voltage rating because the designed pre-matching circuit is located between the limiter and the preamplifier.

Unfortunately, the electrical impedances of ultrasound transducers vary with respect to the frequency, such that it is very challenging and complex to match broadband frequency ranges between resonant and anti-resonant frequency ranges [13,16]. Currently developed matching circuits are designed to match the center (operating) frequency of the transmitter and ultrasound transducer [1,5]. These matching-circuit topologies are widely used in radio frequency (RF)/wireless/communication instrumentations, which must be operated in narrow-band frequency ranges because of the frequency-band selection in certain applications [22,23,24]. 

In addition, for high-frequency transducers, the absolute values of the frequency range between the resonant and anti-resonant frequency ranges are wider than those of low-frequency transducers [13]. Therefore, they might decrease the sensitivity or bandwidth of the high-frequency ultrasound transducers and lower the image resolutions of the ultrasound instruments. Hence, the developed pre-matching circuits are designed to match the electrical impedances of ultrasound transducers with the wide frequency ranges between the resonant frequency and anti-resonant frequency ranges and improve the amplitude and bandwidth of the high-frequency ultrasound transducers. 

This paper will proceed as follows. Section 2 will describe how to construct the designed pre-matching circuits with a resistor-diode limiter, preamplifier, and ultrasound transducer. The impedance matching concept of the series inductor, a parallel capacitor with a series inductor, and wideband pre-matching circuits will be analyzed based on the circuit model; therefore, the specific values of the components in the pre-matching circuits will be determined. Section 3 will show and compare the pulse-echo measurement results of the developed pre-matching circuits with an ultrasound transducer. In the pulse-echo measurement results, peak-to-peak voltage, −6 dB bandwidth, and harmonic distortions of the ultrasound transducer will be compared to further describe the operating mechanisms of the developed pre-matching circuits. Section 4 will summarize the concept of the pre-matching circuit, the motivation of the research, and measurement results, and describe the future work of this paper.

## 2. Materials and Methods

**Figure 1:**
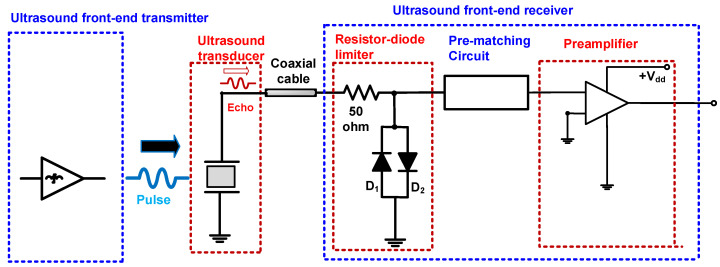
Block diagram of an ultrasound transducer with ultrasound front-end transmitter, receiver, preamplifier, and pre-matching circuit.

Figure 1 illustrates a brief concept of the block diagram of the ultrasound transducer with a front-end transmitter and a front-end receiver that consists of a resistor-diode limiter, pre-matching circuit, and preamplifier.

The resistor-diode limiter contains a 50 Ω series resistor shunt with parallel diodes (D_1_ and D_2_). It is typically used to block unwanted single or multi-cycle pulsed signals generated by the front-end transmitter, e.g., pulse generators or power amplifiers. The preamplifier is used to amplify the weak echo signals obtained from the ultrasound transducers [25]. The pre-matching circuit is located between the resistor-diode limiter and the preamplifier, to improve the performance of the ultrasound transducers. Between the ultrasound transducer and the front-end receiver, a short coaxial cable is used to minimize the signal distortion of the ultrasound transducer’s echo signal [26].

**Figure 2:**
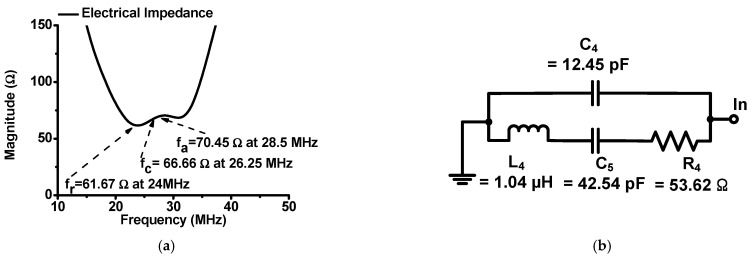
(**a**) Measured magnitude of the electrical impedance, and (**b**) equivalent circuit model of the high-frequency ultrasound transducer.

Figure 2 shows the measured magnitude of the electrical impedance and the equivalent circuit model of the high-frequency ultrasound transducer using an impedance analyzer (E4991A, Agilent Technology, Santa Clara, CA, USA). In Figure 2a, the center frequency (f_c_) of the ultrasound transducer is the average of the combined resonant and anti-resonant frequencies (f_r_ and f_a_) [27]. The matching-circuit structure is the Butterworth model, which was used for the ultrasound transducer model [1,5,22]. There are three equivalent circuit models of the ultrasound transducer, the Mason, KLM, and Butterworth models. In the Mason and KLM models, there is a negative capacitance value in the transformer part because this value was estimated from the ultrasound transducer [5]. However, there are no negative resistance, capacitance, or inductance values in the Butterworth model [1]; therefore, the Butterworth model will be more appropriate when integrating the components of the equivalent circuit model into the complex circuit design tool. Integrating the equivalent circuit model with ultrasound transducers and ultrasound systems into one design tool could allow the engineers to adjust the performance of the device and system efficiently.

As shown in Figure 2b, the Butterworth model of the high-frequency ultrasound transducer is composed of the capacitance (C_4_) with an additional inductor (L_4_), capacitor (C_5_), and resistor (R_4_) [5]. The measured electrical impedances of the resonant-, center-, and anti-resonant-frequencies of the ultrasound transducers were 61.67 Ω at 24 MHz, 66.66 Ω at 26.25 MHz, and 70.45 Ω at 28.5 MHz, respectively.

**Figure 3:**
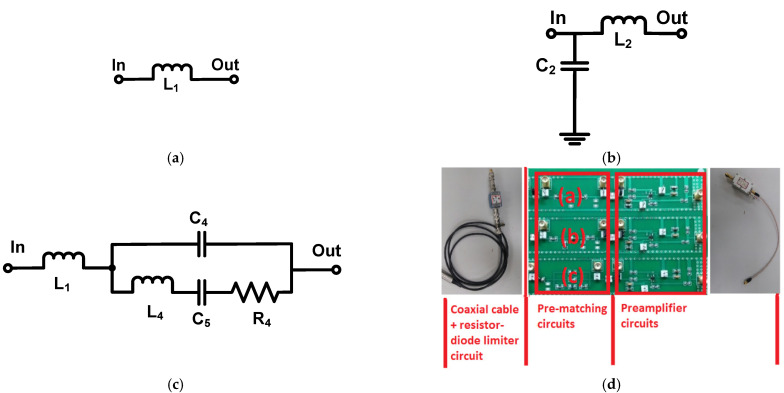
Schematic diagram of: (**a**) series inductor, (**b**) parallel capacitor with series inductor, (**c**) wideband pre-matching circuits, and (**d**) implemented printed circuit board.

Figure 3 shows the circuit schematics of the pre-matching circuits for the receivers. The printed circuit board of the preamplifier, pre-matching, and resistor-diode–limiter circuits was fabricated with a two-layer printed circuit board. The pre-matching circuits were utilized to match the electrical impedances at the center frequency of the ultrasound transducer, thereby improving the sensitivity of the ultrasound transducers. High-frequency ultrasound transducers can be effective when using a pre-matching circuit because they typically have low sensitivity owing to the limited size of piezoelectric materials. Figure 3a–c show the schematic diagrams of the series inductor, the parallel capacitor with a series inductor, and wideband pre-matching circuits. Figure 3d shows the implemented printed circuit board of the pre-matching circuits.

**Figure 4:**
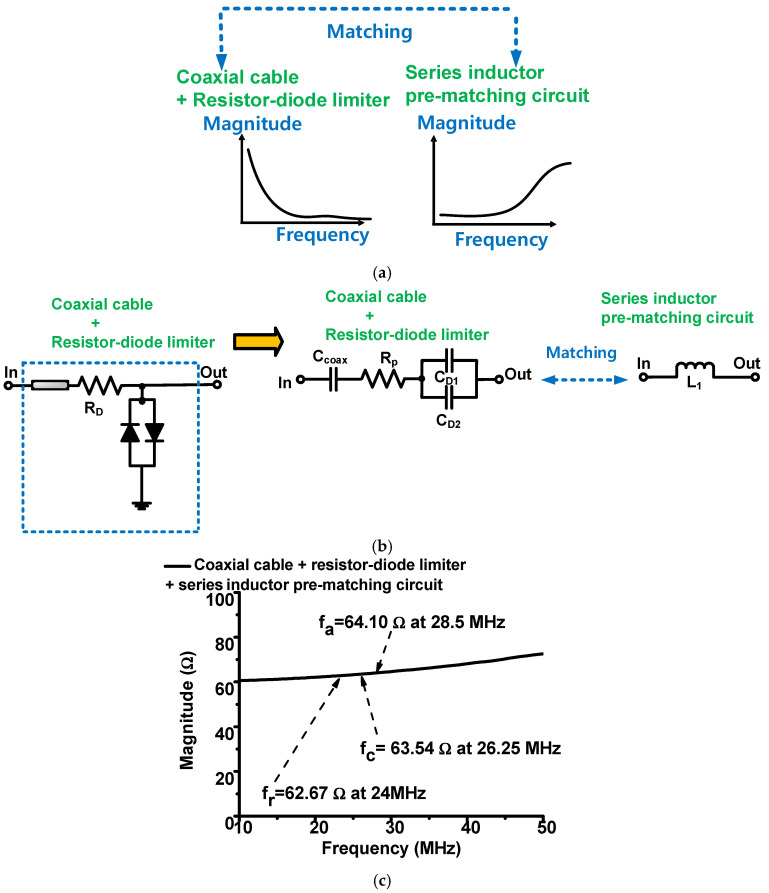
(**a**) Concept of impedance matching; (**b**) equivalent circuit model with a series-inductor circuit; (**c**) measured electrical impedances.

Figure 4a describes the concept of impedance matching between the resistor-diode limiter and series-inductor pre-matching circuit. Figure 4b shows the equivalent circuit model of the resistor-diode limiter resonated out with a series-inductor circuit. Figure 4c shows the measured electrical impedances of the resistor-diode limiter with a series-inductor pre-matching circuit.

As shown in Figure 4a, a series-inductor pre-matching circuit was added to match the resistor-diode limiter with the coaxial cable because the coaxial cable could affect the performance of the high-frequency ultrasound transducers [13,28]. As shown in Figure 4b, the series-transformed diode capacitances and coaxial cable capacitances should be resonated out with a series inductor at the center frequency of the high-frequency ultrasound transducer (w_c_). The inductor with a capacitor is resonant in the center frequency of the transducer. Therefore, the series-inductor value was obtained as follows [29]:

(1) $${\left(w_{c}\right)}^{2}=\frac{1}{L_{1}(C_{D1}+C_{D2}+C_{\mathrm{coax}})}=>{\;L}_{1}=\frac{1}{4\pi^{2}f_{c}{}^{2}(C_{D1}+C_{D2}+C_{\mathrm{coax}})}$$

where w_c_ is 2πf_c_; C_D1_ and C_D2_ are the series-transformed diode capacitances (D_1_ and D_2_), respectively, of the resistor-diode limiter; C_coax_ is the capacitance of the coaxial cable multiplied by the foot length.

Therefore, a series-inductance value (L_1_ = 18 nH, Coilcraft Inc., Cary, IL, USA) was selected using Equation (1), even though the calculated inductance value is 18.2 nH, as the manufacturer does not provide the exact value. Figure 4c shows the measured electrical impedances of the resistor-diode limiter and the series-inductor pre-matching circuit. The electrical impedances of the resonant-, center-, and anti-resonant-frequencies of the resistor-diode limiter with a series-inductor pre-matching circuit were 62.67 Ω at 24 MHz, 63.54 Ω at 26.25 MHz, and 64.10 Ω at 28.5 MHz, respectively. Therefore, the series-inductor pre-matching circuit both resonates with the resistor-diode limiter and matches the ultrasound-transducer impedance at the center frequency because the differences in the electrical impedances at the center frequencies are less than 3.12 Ω, which is a small value.

**Figure 5:**
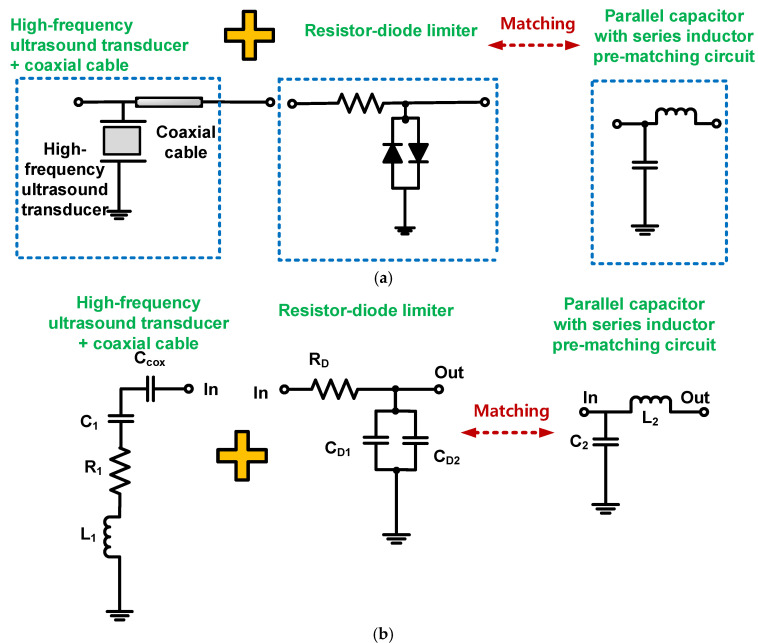
(**a**) Concept of impedance matching and (**b**) combined equivalent circuit model between the transducer, limiter, and parallel capacitor with a series-inductor pre-matching circuit.

Figure 5 shows the impedance transformation model of the high-frequency ultrasound transducer. Figure 5a shows the concept of the equivalent-circuit model of a high-frequency ultrasound transducer to be combined with the equivalent-circuit model of the resistor-diode limiter, using the impedance-transformation model [22,30]. Figure 5b shows the combined equivalent-circuit model of a high-frequency ultrasound transducer and the resistor-diode limiter to calculate the component values of the pre-matching circuit of the parallel capacitor with a series-inductor circuit. We obtained the following resistance (R_1_ = 106.93 Ω), capacitance (C_1_= 1.89 nF), and inductance (L_1_ = 1.02 μH) values in the transformation model, as shown in Figure 5b.

**Figure 6:**
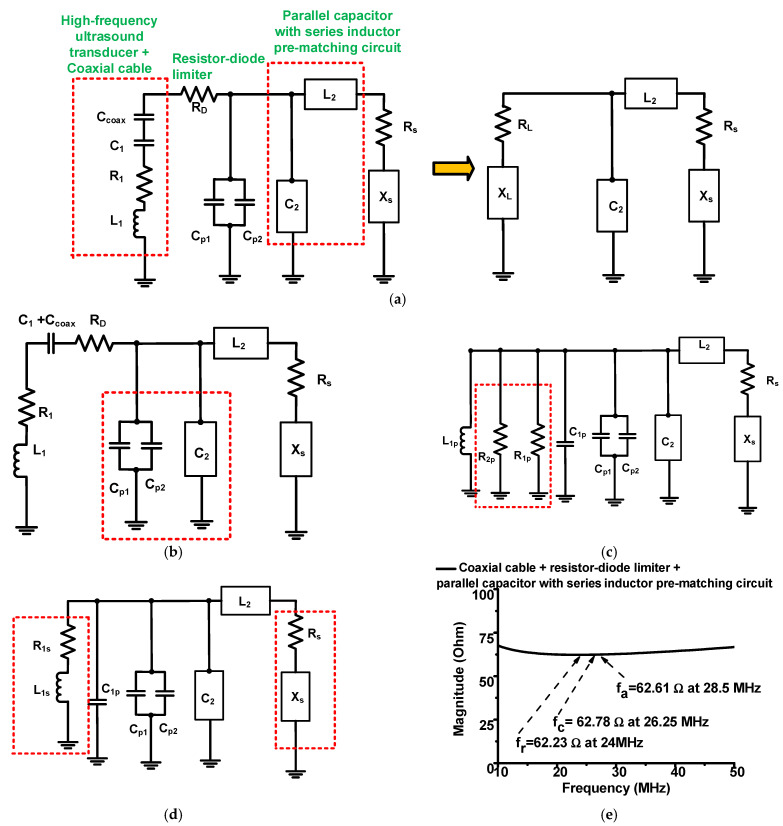
(**a**) Impedance conversion of the transducer with the limiter and the parallel capacitor with a series-inductor pre-matching circuit; the series-parallel impedance conversion of the: (**b**) resistor-diode limiter and (**c**) transducer; (**d**) the parallel-series impedance conversion of the transducer; (**e**) measured impedance of the limiter with a parallel capacitor with a series-inductor pre-matching circuit.

Figure 6 shows how to obtain the values of the parallel capacitor with a series-inductor pre-matching circuit with a high-frequency ultrasound transducer. Figure 6a shows the impedance transformation model of the high-frequency ultrasound transducer with a resistor-diode limiter and the parallel capacitor with a series-inductor pre-matching circuit. Figure 6b,c show the general series-parallel impedance conversion of the resistor-diode limiter and the high-frequency ultrasound transducer, respectively. Figure 6d shows the parallel-series conversion of the high-frequency ultrasound transducer. Figure 6e shows the measured electrical impedance of the resistor-diode limiter with a parallel capacitor with a series-inductor pre-matching circuit. Using a series-parallel or parallel-series transformation, we need to convert this circuit model to another circuit model to obtain the values of the parallel capacitor with a series-inductor pre-matching circuit.

According to the general series-parallel transformation theory, the general parallel resistor (R_p_) could be obtained using the general series resistor (R_s_) with a general series capacitor (R_s_), as shown in Equation (2), and the general parallel capacitor (C_p_) could be obtained using the general series capacitor (C_s_) with the general series resistor (R_s_), as shown in Equation (3) [29,31].

(2) $$R_{p}=R_{s}(1+{\left(2\pi f_{c}C_{s}R_{s}\right)}^{2}$$

(3) $$C_{p}=C_{s}\left(1+\frac{1+{\left(2\pi f_{c}C_{s}R_{s}\right)}^{2}}{{\left(2\pi f_{c}C_{s}R_{s}\right)}^{2}}\right)$$

Using the general series-parallel transformation method mentioned above, the general parallel resistor (R_P_) is the parallel resistor (R_1P_). The general series resistor (R_s_) is the resistor-diode limiter (R_D_). The general series capacitor (C_s_) is a combination of the capacitor in the equivalent circuit model of the transducer (C_1_) and the capacitor of the coaxial cable (C_coax_). Therefore, the resistor in the resistor-diode limiter (R_D_) and the capacitor in the equivalent circuit model of the transducer (C_1_) were converted into the parallel resistor (R_1p_) and capacitor (C_1p_), shown in Figure 6b [24,29,32].

(4) $$R_{1p}=R_{D}(1+{\left(2{\pi f}_{c}(C_{1}+C_{\mathrm{coax}})R_{D}\right)}^{2}\;C_{1p}=C_{1}\left(1+\frac{1+{\left(2{\pi f}_{c}(C_{1}+C_{\mathrm{coax}})R_{D}\right)}^{2}}{{\left(2{\pi f}_{c}(C_{1}+C_{\mathrm{coax}})R_{D}\right)}^{2}}\right),$$

where R_1_, C_1_, and L_1_ are the resistance, capacitance, and inductance, respectively, in the impedance-transformation model. 

According to the general parallel-series transformation theory, the general parallel resistor (R_p_) could be obtained using the general series resistor (R_s_) with the general series inductor (L_s_), as shown in Equation (5), and the general parallel capacitor (C_p_) could be obtained using the general series capacitor (C_s_) with the general series inductance (L_s_) [31].

(5) $$R_{p}=R_{s}(1+{\left(\frac{2{\pi f}_{c}L_{s}}{R_{s}}\right)}^{2}$$

(6) $$C_{p}=C_{s}\left(1+\frac{1}{{\left(\frac{2{\pi f}_{c}L_{s}}{R_{s}}\right)}^{2}}\right)$$

Using the general parallel-series transformation method mentioned above, the general parallel resistor (R_P_) is the parallel resistor (R_2P_); the general series resistor (R_s_) is the resistor (R_1_) in the transducer; the general series inductor (L_s_) is the inductor in the transducer (L_1_).

(7) $$R_{2p}=R_{1}(1+{\left(\frac{2{\pi f}_{c}L_{1}}{R_{1}}\right)}^{2}{\;L}_{1p}=L_{1}\left(1+\frac{1}{{\left(\frac{2{\pi f}_{c}L_{1}}{R_{1}}\right)}^{2}}\right)$$

Therefore, we obtained a series resistor and inductor (R_1s_ and L_1s_) to calculate the values of the parallel capacitor with a series-inductor pre-matching circuit. The resistor (R_1s_) and the inductor (L_1s_) are shown in Figure 6c. Using the series-parallel transformation method from Equations (5) and (6), we also obtained a series resistor and inductor (R_1s_ and L_1s_) to calculate the values of the parallel capacitor with a series-inductor pre-matching circuit in Equation (8). The resistor (R_1s_) and the inductor (L_1s_) are shown in Figure 6d.

(8) $$R_{1s}=\frac{\left(R_{1p}//R_{2p}\right)}{\left(1+{\left(\frac{2{\pi f}_{c}L_{1p}}{R_{1p}//R_{2p}}\right)}^{2}\right)}{\;L}_{1s}=\frac{L_{1p}}{\left(1+{\left(\frac{2{\pi f}_{c}L_{1p}}{R_{1p}//R_{2p}}\right)}^{2}\right)}$$

where R_1p_ and L_1p_ are, respectively, the parallel resistor and inductor after processing the general parallel-series transformation.

By equating the real and imaginary parts of the combined equivalent circuit model and the parallel capacitor with a series-inductor pre-matching circuit, as shown in Figure 6d, we acquired the values of the parallel capacitor with a series-inductor pre-matching circuit.

Using the dual-elements transformation network [33], the general intermediate capacitor could be obtained by two source and load resistors (R_1s_ and R_s_) with the general inductor (L_1s_); the general intermediate inductor (L_2_) could be obtained by two source and load resistors (R_1s_ and R_s_) and the general inductor (L_1s_) if the load resistor is smaller than the source resistor.

From the Equations (9) and (10), the general intermediate capacitor is a combination of four capacitors (C_2_, C_1P_, C_P1_, and C_P2_). Therefore, the calculated values of the component (C_2_ = 980 pF and L_2_ = 220 nH) are obtained from the Equations (9) and (10), such that we can select the values of the component (C_2_ = 1000 pF and L_2_ = 220 nH) provided by the manufacturer to match the combined equivalent-circuit model of the high-frequency ultrasound transducer.

(9) $$\;\;C_{2}=\frac{R_{S}-{\;R}_{1s}}{2{\pi f}_{c}\left(R_{s}\left(2{\pi f}_{c}L_{1s}\right)+R_{1s}\left(2{\pi f}_{c}L_{s}\right)\right)}-(C_{1p}+C_{p1}+C_{p2})$$

(10) $$L_{2}=\frac{\sqrt{R_{s}\left(R_{1s}-R_{S}\right)+\frac{R_{s}}{R_{1s}}{\left(2{\pi f}_{c}L_{1s}\right)}^{2}}}{2{\pi f}_{c}}$$

As shown in Figure 6e, the electrical impedance of the parallel capacitor with a series-inductor pre-matching circuit (62.78 Ω) was similar to that of the high-frequency ultrasound transducer at the center frequency (66.66 Ω). Because the difference in the electrical impedances at the center frequencies is less than 3.88 Ω, the electrical impedances of the high-frequency ultrasound transducer and the pre-matching circuit with a resistor-diode limiter and coaxial cable are well-matched.

**Figure 7:**
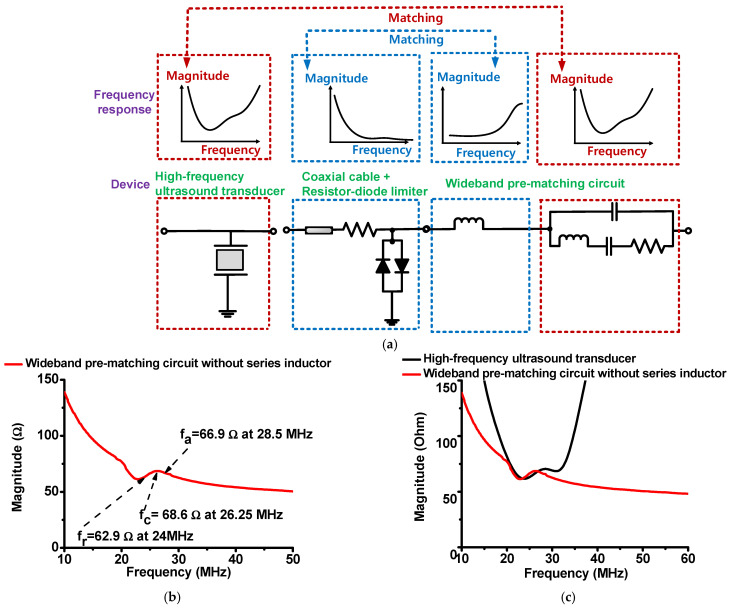
(**a**) Concept of impedance matching for wideband pre-matching circuit. Measured electrical impedances of the wideband pre-matching circuit: (**b**) without a series inductor and (**c**) with the transducer.

Figure 7a demonstrates how to obtain the developed wideband pre-matching circuit between the transducer, resistor-diode limiter, and wideband pre-matching circuit. Figure 7b,c show the measured electrical impedances of the wideband pre-matching circuit and a comparison of the electrical impedances of the high-frequency ultrasound transducer with a wideband pre-matching circuit. In the circuit, the series inductor needs to resonate with the diode capacitance in the resistor-diode limiter. The other components, which consist of a parallel capacitor with a series inductor, capacitor, and resistor, are matched with a high-frequency ultrasound transducer.

In Figure 2a, the electrical impedance of the center frequency (f_c_) was 66.66 Ω at 24 MHz, which is not 50 Ω. In addition, the electrical impedances of the resonant- and anti-resonant-frequencies (f_r_ and f_a_) were, respectively, 66.66 Ω at 26.25 MHz and 70.45 Ω at 28.5 MHz, which varied with respect to the frequency. In RF/communication systems, the electrical-impedance matching condition should match 50 Ω; hence, we cannot utilize their approach exactly [22].

In Figure 2b, the wideband pre-matching circuit is composed of an inductor-capacitor-resistor (C_4_ = 12 pF, L_4_ = 1 μH, C_5_ = 43 pF, and R_4_ = 51 Ω) with an additional series inductor (L_1_ = 4.7 μH) provided by the manufacturer. These values are similar to the equivalent-circuit model of the high-frequency ultrasound transducer (C_4_ = 12.45 pF, L_4_ = 1.04 μH, C_5_ = 42.54 pF, and R_4_ = 53.62 Ω). As shown in Figure 3c, the matching inductor value (L_1_) was selected to resonate out the diode capacitance of the resistor-diode limiter at the center frequency of the ultrasound transducer, using Equation (1). The diode capacitances also resonated with the series inductance in the wideband pre-matching circuit at the center frequency of the ultrasound transducer.

**Figure 8:**
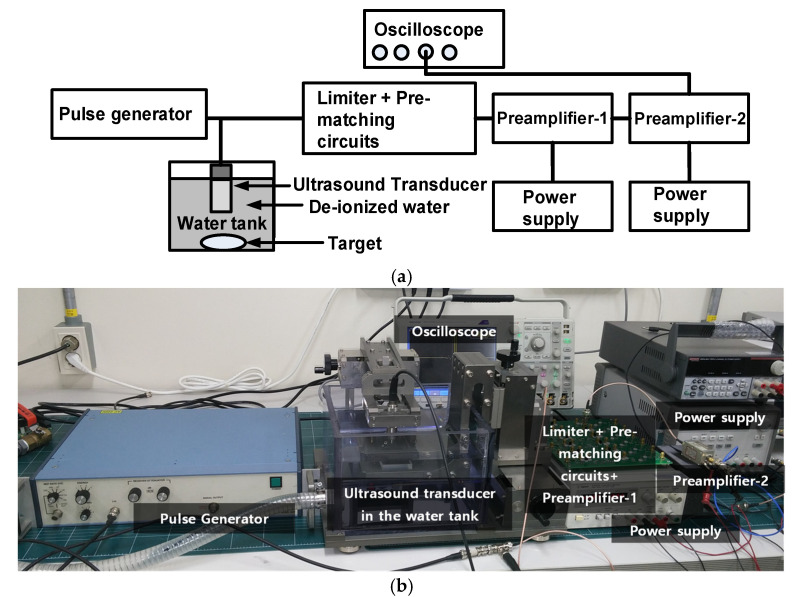
(**a**) Block diagram and (**b**) photo of the pulse-echo response measurement setup.

The components of the wideband pre-matching circuit have values similar to the measured equivalent model of the ultrasound transducer between the resonant and anti-resonant frequency ranges, only because the exact value could not be obtained from the manufacturer. Therefore, the electrical impedance graph of the ultrasound transducer differs slightly from that of the wideband pre-matching circuit. The electrical impedances of the resonant-, center-, and anti-resonant-frequencies of the ultrasound transducers and wideband pre-matching circuit were 61.67 Ω and 62.9 Ω at 24 MHz, 66.66 Ω and 68.6 Ω at 26.25 MHz, and 70.45 Ω and 66.9 Ω at 28.5 MHz, respectively. Therefore, the maximum differences in the electrical impedances at the resonant-, center-, and anti-resonant-frequencies were less than 3.6 Ω. This approach is simple compared to the approach for a parallel capacitor with a series-inductor pre-matching circuit. It is also expected to obtain electrical impedance matching in the wideband frequency ranges. Figure 8a,b show the block diagram and photo of the pulse-echo measurement with ultrasound transducer.

## 3. Results

**Figure 9:**
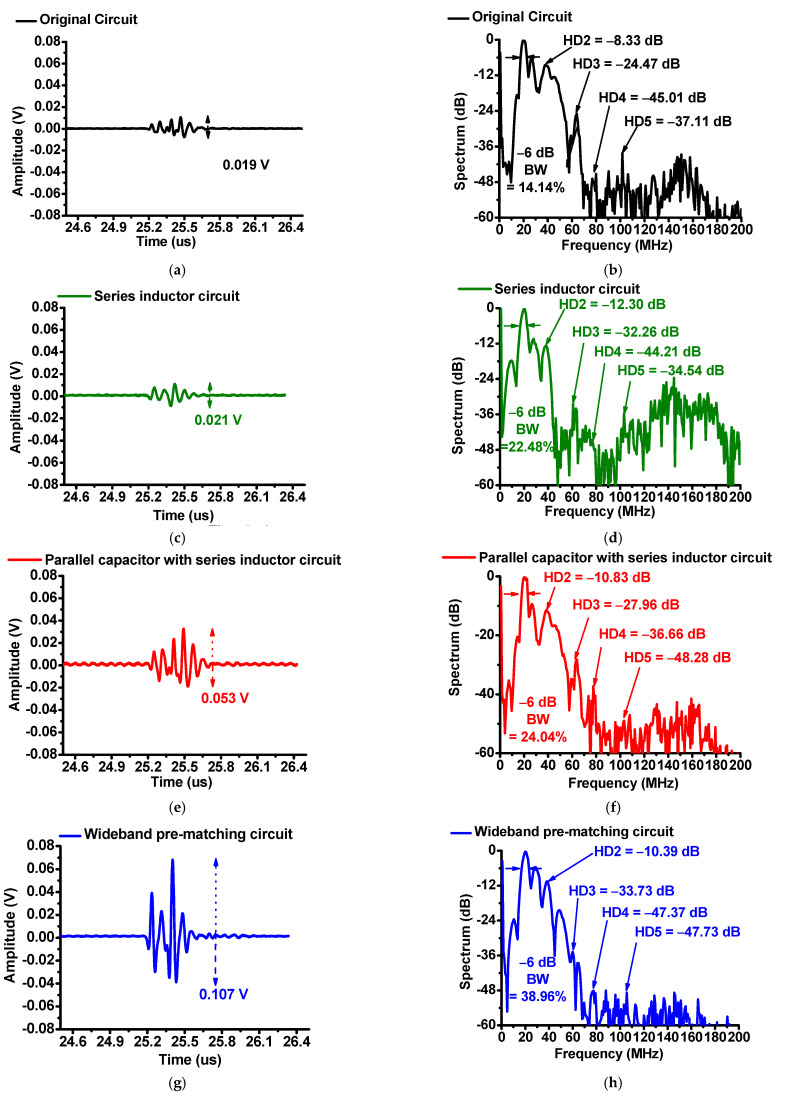
Pulse-echo responses of the ultrasound transducer: (**a**) The amplitude and (**b**) spectra of the transducer; (**c**) the amplitude and (**d**) spectra when using a series-inductor circuit; (**e**) the amplitude and (**f**) spectra when using a parallel capacitor with a series-inductor circuit; (**g**) the amplitude and (**h**) spectra when using a wideband pre-matching circuit.

Figure 9 shows the comparison data when using the original circuit, a series inductor, a parallel capacitor with a series inductor, and wideband pre-matching circuits in the pulse-echo response. As shown in Figure 9a,c,e,g, the peak-to-peak amplitudes of the echo signal when using the series inductor, the parallel capacitor with series-inductor pre-matching circuits, and wideband pre-matching circuit were, respectively, 1.12-, 2.72-, and 5.63-times those of the original circuit without using pre-matching circuits. Figure 9b,d,f,h show the spectra data when using the series inductor, the parallel capacitor with series-inductor pre-matching circuits, and wideband pre-matching circuit, respectively.

As shown in Figure 9b,d,f,h, the −6 dB bandwidths of the original circuit, series-inductor pre-matching, parallel capacitor with series-inductor pre-matching, and wideband pre-matching circuits were 14.14, 22.48, 24.04, and 38.96%, respectively. The spectra of the echo signal, when using the series-inductor pre-matching circuit, the parallel capacitor with a series-inductor pre-matching circuit, and wideband pre-matching circuits, were 4.58, 7.73, and 31.01% wider than those of the original circuit, respectively.

The total harmonic distortions (THDs), when using the original circuit, series-inductor pre-matching, the parallel capacitor with a series-inductor pre-matching, and wideband pre-matching circuits, were −16.44, −24.43, −21.46, and −20.71 dB, respectively. The THDs when using the series inductor, the parallel capacitor with a series inductor, and the wideband pre-matching circuits were, respectively, improved by 7.98, 5.02, and 4.26 dB, compared to when using the original circuit. Compared to the series-inductor pre-matching circuit, the THDs of the parallel capacitor with series inductor pre-matching and the wideband pre-matching circuits were worse, because the additional inductor, capacitor, and resistor components might generate unwanted harmonics. Therefore, the highest THD was achieved when using the series-inductor circuit; Therefore, we can confirm that the developed wideband pre-matching circuit can help improve the amplitudes and bandwidths of high-frequency ultrasound transducers.

Table 1 summarizes the measurement results of the pulse-echo responses of the ultrasound transducer using the series inductor, the parallel capacitor with a series inductor, and wideband pre-matching circuits. The series-inductor pre-matching circuit is very simple; however, the performance of the peak-to-peak voltage, bandwidth, and harmonic distortion could be enhanced a little. Therefore, this approach could be useful if the size of the printed circuit board is very limited. The parallel capacitor with the series inductor pre-matching circuit could be a better choice if the sensitivity and bandwidth of the ultrasound transducer affect the ultrasound system performance. However, mathematical analysis with the ultrasound transducer model and resistor-diode limiter would be required to calculate the discrete components of the parallel capacitor with a series inductor pre-matching circuit. The wideband pre-matching circuit is the optimal option because the peak-to-peak voltage and bandwidth of the echo signals could be improved; however, there is enough space required for this to be implemented on the printed circuit board and harmonic distortion is not a critical issue for its ultrasound-signal processing task.

Table 2 shows the comparison data of the proposed approach with other scholars’ work for impedance-matching topology used in ultrasound transducer applications. As shown in Table 2, the matching circuits are composed of the inductor with capacitor matching, the capacitor with inductor matching, or T- or Pi-network. The T- or Pi-network matching circuits are combination circuits of the inductor with capacitor matching and the capacitor with inductor matching circuits [19,20]. The target parameter of some matching circuits is the amplitude only or amplitude and bandwidth together.

## 4. Conclusions

The electrical impedances of ultrasound transducers should vary with respect to the frequency, such that the electrical impedances of the resonant-, center-, and anti-resonant-frequencies of the ultrasound transducers differ accordingly. This makes it difficult for them to match in wide frequency ranges. The typical approach for using pre-matching circuits was to match them with the electrical impedances at the center frequency of the ultrasound transducers; however, this sometimes led to lower sensitivity or smaller bandwidths.

The bandwidth of high-frequency ultrasound transducers is critical issue because the parameter is related to the transducer’s performance. Additionally, matching circuit topologies were used to match with the ultrasound transducers on the transmitter sides. Therefore, all components used in the matching circuits must be tolerated in high-voltage operations. This can affect the performance of the power amplifiers or pulse generators, which are difficult to control from the perspective of the design level.

The approach provided matching conditions at the receiver side, thereby providing more freedom to select the components in the matching circuits. The matching condition with coaxial cables and a resistor-diode limiter might be complicated to design. Therefore, we proposed novel wideband pre-matching circuits to increase the amplitudes and bandwidths of the echo signals generated by high-frequency ultrasound transducers.

We compared developed pre-matching circuits, e.g., a series inductor and a parallel capacitor with series-inductor pre-matching circuits, with the developed wideband pre-matching circuit. The peak-to-peak amplitudes of the echo signal when using the series inductor, the parallel capacitor with a series-inductor, and wideband pre-matching circuits were, respectively, 1.10-, 2.78-, and 5.63-times higher than when using the original circuit. Additionally, the −6 dB bandwidths of the echo signals when using the series inductor, the parallel capacitor with a series-inductor pre-matching, and wideband pre-matching circuits were 59.02%, 70.00%, and 175.52% wider, respectively, compared to when using the original circuit.

The series inductor pre-matching circuit is very simple so it is widely used because it is useful to improve the signal quality of the echo signals a little and the size of the system is small. The parallel capacitor with a series inductor pre-matching circuit could be good if the sensitivity and bandwidth of the transducer need to be improved; however, complex mathematical analysis with the circuit background needs to be utilized to calculate the components of the parallel capacitor with a series inductor pre-matching circuit. The wideband pre-matching circuit could be a better choice because the peak-to-peak voltage and bandwidth of the echo signals could be improved. However, the integrated circuit design process is preferable with this approach and harmonic distortion is not an important parameter issue to be implemented.

Therefore, we demonstrated that the developed wideband pre-matching circuit could improve the amplitude and bandwidth performance of high-frequency ultrasound transducers using pulse-echo responses. This could be very useful for high-frequency ultrasound transducers with low amplitude. In addition, the design of the wideband pre-matching circuit was simpler to implement than the parallel capacitor with a series-inductor pre-matching circuit.

In the future, the proposed pre-matching circuit will be applied to ultrasound transducers, which have a very low amplitude. The ultrasound transducers used in high-frequency intravascular applications have very small sensitivity. Due to the size limitation of the intravascular structure, the element size of the ultrasound transducer is small, resulting in obtaining wide bandwidth comprising the amplitude. With the help of the newly proposed pre-matching circuit, the amplitude and bandwidth of the transducer for intravascular applications could be enhanced. There are fewer burdens of the ultrasound system design, which could improve the signal-to-noise ratio. However, these components need to be implemented in the integrated circuit due to several discrete components if a size limitation of the intravascular structure is required. Therefore, the pre-matching circuit could be integrated with the ultrasound transmitter and receiver in intravascular applications.

## Figures and Tables

**Table 1:** Summary of the pulse-echo response measurements of the ultrasound transducer.

	Original	Series Inductor	Parallel Capacitor with Series Inductor	Wideband
V_p-p_ (V)	0.019	0.021	0.053	0.107
BW (%)	14.14	22.48	24.04	38.96
HD2 (dB)	−8.33	−12.30	−10.83	−10.39
HD3 (dB)	−24.47	−32.26	−27.96	−33.73
HD4 (dB)	−45.01	−44.21	−36.66	−47.37
HD5 (dB)	−37.11	−34.54	−48.28	−47.73
THD (dB)	−16.44	−24.43	−21.46	−20.71

V_p-p_ and BW represent the peak-to-peak voltage and −6 dB bandwidth, respectively.

**Table 2:** Comparison data of the proposed pre-matching circuit with other scholars’ work.

	[15]	[16]	[17]	[18]	[19]	[20]	This Work
Center or Resonant Frequency	0.8 MHz	60.66 kHz	36.1 MHz/46.8 MHz	0.3 MHz	250, 350, 450 kHz	1.5 MHz	24 MHz/26.25 MHz/28.5 MHz
Topology	Inductor with Capacitor Matching	Capacitor with Inductor Matching	T-network Matching	T-network Matching	T-network Matching	Pi-network/ T-network Matching	Inductor/Capacitor with Inductor/Wideband Matching
Target Parameters	Amplitude	Amplitude	Amplitude/Bandwidth	Amplitude	Amplitude	Amplitude	Amplitude/Bandwidth
Application	High Impedance Transducer	Piezoelectric Ultrasonic Transducer	Piezoelectric Ultrasonic Transducer	Piezoelectric Ultrasonic Transducer	Piezoelectric Ultrasonic Transducer	Capacitive Micromachined Ultrasonic Transducer	Piezoelectric Ultrasonic Transducer

## Data Availability

The data presented in this study are included within the article.
